# Thankful employees: The manifestation of gratitude at work during a pandemic in South Africa

**DOI:** 10.3389/fpsyg.2022.941787

**Published:** 2022-07-22

**Authors:** Lusanda Sekaja, Courtney A. Tully, Senzile Mahlangu, Katya de Freitas, Lihle N. Tyelbooi, Bonisiwe P. L. Mjojeli, Masase E. Mokhethi, Tshegofatso Mabitsela

**Affiliations:** Department of Industrial Psychology and People Management, University of Johannesburg, Johannesburg, South Africa

**Keywords:** gratitude, workplace gratitude, generic qualitative approach, positive psychology, South Africa, pandemic, COVID-19

## Abstract

Gratitude or the appreciation of being given something of value, is an important element in positive emotions within positive psychology. Gratitude has been linked to wellbeing and gratitude in the workplace is positively associated with constructs such as performance and organizational citizenship behavior. The pandemic brought on many negative experiences but employees could still find things to be grateful for during this time. The purpose of the study was to understand what aspects of work and the organization employees were grateful for during the pandemic. A generic qualitative approach was used. Participants were sourced from various industries in South Africa using purposive sampling. Data were gathered through 21 semi-structured interviews of working people in South Africa. Braun and Clarke’s thematic analysis revealed five themes, namely, (1) gratitude for no negative work-life changes; (2) gratitude for a caring workplace; (3) gratitude for a new way of working; (4) gratitude for the ability to put oneself first; and (5) gratitude for having resilience, optimism and spirituality as a psychological buffer. Managers should deliberately engage in behaviors that will bring about gratitude from their employees. Employees should reflect on the positive things at work that they are thankful for as a way of enhancing gratitude and thereby, wellness, performance, and commitment. The study combines existing knowledge on gratitude during the pandemic with gratitude in the workplace.

## Introduction

COVID-19 has had a well-documented negative impact on the world, and South Africa is no exception. South Africa felt this impact from psychological, social, medical, educational, and economic points of view (Arndt et al., 2020; Bonaccorsi et al., 2020; Sekyere et al., 2020; Sun et al., 2020; Álvarez-Iglesias et al., 2021), through an imposed lockdown (Ramaphosa, 2020, 2022), a lack of social interaction with loved ones (Greyling et al., 2021), a decline in physical and mental wellbeing (Álvarez-Iglesias et al., 2021), illness and loss of life (Ramaphosa, 2020), inaccessibility of online learning and challenges in remote instruction for learners (Gumede and Badriparsad, 2022), forced virtual work (Leask and Ruggunan, 2021), and permanent closure of businesses and/or loss of work (Mhlanga and Moloi, 2020; Statistics South Africa, 2020; Van Lancker and Parolin, 2020; Ramaphosa, 2022).

The impact of the latter can still be felt strongly as life returns toward normalcy. Despite these occurrences, some individuals were able to see the silver lining in the pandemic-induced way of life, look beyond their misfortunes, and find things to be grateful for.

Emmons (2004) defined gratitude as “a sense of thankfulness and joy in response to receiving a gift, whether the gift can be a tangible benefit from a specific other or a moment of peaceful bliss evoked by natural beauty” (p. 554). According to Bono et al. (2020), individuals can experience subjective wellbeing, which could have helped them cope with the challenges of the pandemic when they viewed the hardship of the pandemic through the lens of gratitude. For example, while the pandemic has caused a disruption to life as people had known it, focusing on positive aspects, such as still having a job, supportive and understanding leadership, and freedom to their workdays while working remotely (Sangoni, 2020), gratitude during times of crisis can help individuals sustain a positive outlook, which serves to energize and motivate them (Fishman, 2020).

## Problem statement

Gratitude has many benefits. Research shows that gratitude has positive relations with the performance of employees (Wang et al., 2020), the productivity of employees (Grant and Wrzesniewski, 2010), job satisfaction (Cortini et al., 2019), and organizational citizenship behaviors (OCBs; Chen et al., 2020). The practice of gratitude has also shown to enhance the wellbeing of employees (Kaplan et al., 2014); Lambert et al. (2009) explained that because individuals who experience gratitude, are more likely to focus their attention on the positives, these individuals will have a greater propensity of managing obstacles they encounter.

Fishman (2020) recommended that during times of crisis (such as the COVID-19 pandemic), people may engage in exercises such as starting a gratitude journal to document all the good things that have happened that they can appreciate in their lives. Fishman (2020) also recommended that individuals re-evaluate past negative experiences for the purpose of identifying their vulnerabilities and appreciating their resilience and ability to adapt. Jans-Beken and Wong (2019) found that when individuals showed existential gratitude (i.e., gratitude for both ease and hardship), they were able to cope during adverse times.

While research predominantly shows that gratitude is inextricably linked to wellbeing, there is a dearth of empirical research showing the negative impact of gratitude. However, early contemplations on the prospect suggest that demonstrating gratitude can sometimes be damaging in certain situations. For example, when an employee has an abusive leader, showing appreciation may have a detrimental effect because it leads the employee to tolerate a situation they should not otherwise accept and thus, in this instance, gratitude is deemed inappropriate and excessive (cf. Card, 2016; Wood et al., 2016). Furthermore, in situations where there are disparities in power, the mutual display of gratitude by individuals could work against the individual with lesser power by unwittingly maintaining as a sense of dependence on the more powerful individual (Ksenofontov and Becker, 2020). Thus exhibiting thankfulness inappropriately to an undeserving other deprives them of the opportunity to recognize and reflect on their negative behavior and therefore make the necessary changes in behavior (Wood et al., 2016).

While there is a plethora of research and commentary on gratitude (e.g., Emmons and McCullough, 2003; Emmons and Mishra, 2011; Davis et al., 2016; Cunha et al., 2019; Yoo, 2020; Nawa and Yamagishi, 2021), gratitude at work (e.g., Cortini et al., 2019; Komase et al., 2019; Chhajer and Dutta, 2021; Komase et al., 2021; Unanue et al., 2021), and gratitude during the pandemic (e.g., Fishman, 2020; Feng and Yin, 2021; Jans-Beken, 2021; Fekete and Deichert, 2022), to the best of the authors’ knowledge, research on gratitude at work during the pandemic remains a lacuna (Youssef-Morgan et al., 2022).

In the context of the pandemic in South Africa, insufficient exploration has been made into developing an understanding of how expressing gratitude manifests positive outcomes (Bono et al., 2020; Fishman, 2020; Shen and Sosa, 2020). Gratitude builds enduring resources (e.g., skills for showing appreciation, social bonds) that function as reserves that can be used in difficult times (Fredrickson, 2004). Research efforts within South Africa should therefore be geared toward exploring the beneficial effects of gratitude during the COVID-19 pandemic.

The South African economy was already vulnerable when the pandemic first struck. In the fourth quarter of 2019, the economy of South Africa shrank by 1.4%, jobs were shed (Statistics South Africa, 2020), and the country’s medical resources were limited (Furtak and Barnard, 2021). With such contextual challenges at play and the advent of a real-world crisis, delving deeper into understanding how gratitude positively manifests itself can help to illuminate how individuals cope with challenging situations (Wood et al., 2007).

To this end, the lack of research into the previously mentioned positive effects associated with the expression of gratitude has motivated the present researchers to carry out this study. With this in mind, the objective for the current study was to investigate the sources of gratitude for South Africans at work during the COVID-19 pandemic.

## Literature review

### Conceptualizing gratitude

Gratitude is a prominent construct within the framework of positive psychology. People’s natural tendency to respond positively to another person’s altruism is characterized by gratitude (Emmons and Stern, 2013). According to Sansone and Sansone (2010), gratitude is concerned with being thankful for those things that one values and that hold meaning for oneself. Grateful individuals give thanks to someone or something for their fortune (Emmons, 2003). It is a state of being appreciative.

Gratitude has been conceptualized as both a trait and state. In its trait form, gratitude refers to an individual’s propensity to show appreciation for the goodwill of others (McCullough et al., 2002) and in its state form, gratitude is about feeling thankful in one particular moment (Emmons and Mishra, 2011). When someone understands and appreciates the benefits they receive from others, they experience gratitude (Emmons and Mishra, 2011). It is therefore possible, especially for those that do not have an affinity for gratitude, to develop it. Merçon-Vargas et al. (2018) identified three characteristics that can be utilized to determine gratitude. They state that the beneficiary must (1) freely accept something beneficial from another person (benefactor), (2) have pleasant thoughts about the other person’s activities, and (3) want to freely reciprocate this benefit. Gratitude, in simple terms, is an emotional state roused when one appreciates obtaining something as a result of someone’s good intentions.

Gratitude has been studied as an attitude, a personality characteristic, a moral virtue, and a coping technique (Mahipalan and Sheena, 2019). For example, Watkins (2013) defined gratitude in three ways:

• as an *emotion* or *mental state*—a person recognizes that they have received a benefit or experienced something good as a result of someone else’s actions;
• as an *affective characteristic*—an individual naturally endowed with a high level of gratitude and will therefore probably be grateful more frequently than others; or
• as a *mood*—gratitude can sometimes be an emotional state that endures over an extended period.

According to various definitions of gratitude, first, a person acknowledges the goodness that they are experiencing; second, they recognize that the source of these good things is external to themselves; finally, a person who benefits from another’s gratitude must be willing to reciprocate it. Two of the strongest predictors of gratitude are the giver’s responsiveness to the needs of the receiver and whether the benefit provided is liked by the receiver (Algoe and Haidt, 2009).

Gratitude has been linked to a number of wellbeing-related outcomes for people, such as mental health, contentment, happiness, a healthy sense of pride, hope, a higher degree of life satisfaction, and reduced stress and depression (Lavelock et al., 2016; Randolph, 2017; Cain et al., 2019; Komase et al., 2019, 2021; Nezlek et al., 2019).

### Gratitude in the workplace

An individual who generally exhibits gratitude in their personal life may not necessarily do so at work (Cain et al., 2019). In the context of the organization, gratitude is seen as the act of being thankful for any work-related benefits and how they may positively impact one’s life (Cain et al., 2019), an individual’s predisposition to experience positive emotions in the context of their profession (Emmons and Stern, 2013), and feeling appreciative in response to their job (Cain et al., 2019). According to Youssef-Morgan et al. (2022), at work, gratitude is conceptualized and operationalized as (1) grateful appraisals of work, (2) gratitude toward colleagues, and (3) a purposeful attitude of gratitude. Work-related outcomes such as job performance and OCBs are also said to be positively correlated with gratitude in the workplace (Cain et al., 2019; Komase et al., 2019). Having demonstrated the beneficial effects of gratitude, organizations can utilize gratitude as a strategy to enhance the wellbeing of employees (Fehr et al., 2017).

Anticipated gratitude also has a bearing on performance. This form of gratitude speaks to how one anticipates that others, such as colleagues and supervisors, may feel grateful for one’s efforts and contributions. For example, Grant and Wrzesniewski (2010) investigated whether the relationship between core self-evaluations and performance was determined by anticipated guilt and gratitude. They found that employees who held high core self-evaluations and experienced anticipated guilt and gratitude were more likely to be high performers. In an experiment by Grant and Gino (2010), it was found that supervisors’ expressions of gratitude increased the input of call center agents. Furthermore, according to Hu and Kaplan (2014), if performance management systems can foster a culture of cooperativeness among team members, these can elicit gratitude.

Gratitude can also foster social bonds and prosocial behavior among colleagues (Cameron and Spreitzer, 2012; Lomas et al., 2014). It is associated with supervisor satisfaction (Hu and Kaplan, 2014) and, according to Fredrickson (2004), social bonds are strengthened through gratitude when individuals give for the sake of benefiting another, rather than expecting something in return. Once a benefit has been given, the giver is motivated to do more for the current and future beneficiary—an important consideration for OCB (Grant and Gino, 2010) and moreover, a culture of reciprocity, teamwork and recognizing the good that others do (Dik et al., 2015). When individuals experience gratitude, they tend to ascribe the benefit they receive as valuable and thus view the act of giving as more altruistic and appraise the giver more positively. This could therefore lead to greater satisfaction with the social aspect of work, that is, with colleagues and supervisors (Smith et al., 1969; Wood et al., 2008b).

Given its link to other organization-related constructs, it can be seen that gratitude is an important player in enhancing the performance of employees and overall health of organizations (Snyder et al., 2014; Fehr et al., 2017).

## Materials and methods

### Research method

We decided that the most suitable way to fulfill the research objectives was to conduct a study using the generic qualitative approach as a research strategy. This strategy is most suitable in studies where the focus is on external, real-world, subjective experiences, rather than inner emotions (Percy et al., 2015). This allowed us to glean individuals’ perspectives of their experiences of gratitude during the pandemic. We adopted an interpretivist research philosophy to understand the value associated with the experiences that made the participants grateful.

### Sampling

Purposive sampling was used to ensure that we spoke to the part of the population that had experiences that brought about gratitude (Campbell et al., 2020). A total of 21 participants took part in the study. It was apparent that data saturation had been reached at 18 participants, but a further three were interviewed to make sure that this was indeed the case. Participants had to be adults residing in South Africa who worked during the COVID-19 lockdown. Table 1 contains the biographical characteristics of the participants.

**TABLE 1:** Characteristics of participants.

Participant	Gender identity	Racial identity	Age	Occupation
P1	Male	Indian	31	Mechanical engineer
P2	Female	Black	35	Journalist
P3	Female	White	56	Self-employed
P4	Female	White	48	Marketing director
P5	Male	White	32	Employed
P6	Female	White	35	Human capital business partner
P7	Female	Black	30	HR administrator
P8	Female	Black	21	Technician
P9	Male	Black	24	Unemployed
P10	Male	Black	26	HRSS administrator and executive assistant
P11	Male	Black	47	Attorney
P12	Female	Black	44	HR assistant
P13	Female	Black	55	Professional nurse
P14	Male	Black	28	Graphic designer
P15	Female	Black	22	Employed
P16	Male	Black	26	Self-employed
P17	Female	Black	32	Employed
P18	Male	Black	23	Employed
P19	Male	Black	39	OD manager
P20	Female	Black	39	Administrative officer
P21	Female	Black	36	Compliance administrator

### Data gathering

We employed semi-structured interviews to gather data for this study, as this was deemed the most appropriate technique. An interview may be understood as a guided or unguided conversation between two people in order to uncover information, ideas, beliefs, and experiences. A semi-structured interview contains predetermined, open-ended questions and allows the interviewer to probe the participant for further explanations on their given answers (Adams, 2015). Semi-structured interviews are conducted conversationally and allow the researcher the freedom to construct additional questions during the interview process to retrieve subjective information from the interviewee.

Each participant was asked the same set of pre-determined questions, and with each individual response, the interviewer asked probing questions for further clarity and exploration (cf. Busetto et al., 2020). The interviews lasted between 30 and 54 min. Examples of questions asked included “Tell me about the aspects of your work that the pandemic has made you grateful for” and “What are the good things you learnt about your colleagues during this time? In what ways did this make you grateful?” The full list of questions can be found in Table 2. Because the interviews took place during lockdown, the interviews were conducted *via* Zoom and Microsoft Teams and were recorded on the same platform (see Saarijärvi and Bratt, 2021).

**TABLE 2:** Interview guide.

Think about yourself as an employee during COVID-19 and the subsequent lockdowns as you answer these questions.
Tell me about the aspects of your work that the pandemic has made you grateful for.
In what other ways did you feel fortunate during this time at work? Why did this make you feel grateful? Do you have another example to give me?
Tell me about the positive lasting changes this pandemic will have on your work.
What positive things did you learn about yourself during this time, as an employee? Is this something you are grateful for? Why?
What positive things did you learn about your colleagues during this time? Is this something you are grateful for? Why?
What did you appreciate about your manager during this time? Why?
Tell me about your resilience at work during this time. Would you say you drew on the resilience during this time for work? Explain how this helped you cope with work during the pandemic.

### Data analysis

Following the chosen research strategy, Braun and Clarke (2006) thematic analysis was deemed a fitting mode of data analysis. First, the interviews were transcribed verbatim from the audio recordings. Next, we read and re-read the transcripts in order to familiarize ourselves with the data. At this point, mental notes were made of the texts of interest and any patterns that were emerging. The text was then selectively coded for data that spoke specifically to the study objectives. Once the text was coded, like codes were banded together and we searched for themes by starting to think about the salient features of those codes. Once we had established themes, we reviewed them, combining some when they shared similar meaning and breaking others apart when the codes were sufficiently different. Next, the themes were given names and each was defined according to what they meant in the context of the data. Finally, the report on the findings was written.

### Rigor

Rigor was maintained in several ways in this research, per the guidelines of Forero et al. (2018). Firstly, credibility was upheld through peer debriefing—the lead researcher communicated with the various co-authors at different stages of the research to ensure alignment. Investigator authority was maintained, as all authors were trained on how to conduct semi-structured interviews. Transferability was maintained through purposive sampling as the participants had to meet specific requirements for inclusion. Dependability was maintained by providing a rich description of the study methods. Lastly, confirmability was ensured through researcher triangulation, as there were multiple authors who all gave input for a higher quality study.

### Ethical considerations

The University of Johannesburg’s Department of Industrial Psychology and People Management Research Ethics Committee granted ethical clearance for the study [Clearance Number IPPM-2020-449(H)]. The participants were informed fully about the nature of the study and their participation, so that they could provide their informed consent. The participants partook in the study out of their own volition, and they were informed that they could withdraw at any time without consequence or needing to give an explanation. Confidentiality was achieved by protecting the identities of the participants and as such, participant codes (P1, P2, P3, and so forth) were used. Data were carefully stored and managed in password-protected computers that were only accessible by the researchers.

## Results

The data analysis yielded five themes, namely, (1) gratitude for no negative work life changes; (2) gratitude for a caring workplace; (3) gratitude for a new way of working; (4) gratitude for the ability to put oneself first; and (5) gratitude for having resilience, optimism and spirituality as a psychological buffer. All the themes are presented below:

### Theme 1: Gratitude for no negative work life changes

Participants were most grateful for not experiencing any major, negative work-related changes. Many people around them had experienced major negative work such as the loss of income and subsequently, their homes, possessions, and lifestyles. Most important for the participants was the fact that they were able to keep their jobs and income which would then allow them to maintain their current lifestyles:

*“I am grateful for having a stable income.”* (P5)

*“I have a home still because a lot people have actually been so badly affected by this pandemic. They have lost their jobs and have had to turn to smaller accommodation or have lost their homes completely.”* (P2)

*“I was fortunate enough to still have my job when the pandemic hit and to still have it currently during the pandemic because a lot of people in the country and globally lost their work.”* (P21)

For other participants, the things that stayed the same and were appreciated tended to be smaller, yet these still played an important role. For example, another participant recognized that apart from having a home, the small things also had a big impact. This included the ability to purchase necessities such as food and warmth.

*“The fact that we had a roof over our heads, we had a plate of food in front of us, we were kept warm through the winter season*…” (P4)

Lastly, others were thankful for not having contracted COVID-19, maintaining good health and life, and thereby still possessing the ability to work:

*“I was not infected by COVID-19, I am still strong and healthy and able to work so that is what I am mostly grateful for.”* (P10)

### Theme 2: Gratitude for a caring workplace

Compassion in organizations did not go unnoticed during this time. Both management and fellow colleagues showed care and support during this time. Management tended to show understanding and removed certain barriers to enable success during this period:

*“So the support that they gave us—it’s not like they were forced to give it, but they gave it willingly. So it just comes out to show that the company cared about us. As much as they are just employers, they cared about us.”* (P15)

Participants detailed gratitude for the support they received from their employer. This support manifested in various ways, such as being provided with the necessary tools to protect participants from contracting the virus. Participants were also grateful for the introduction of work-from-home policies to lessen their chances of contracting the virus.

*“We were mobilized by our company to work from home; everything was supplied for us by them. We have great support structures that were given to us from work, basically, like wellness or, you know, support, if you needed ‘Hello Doctor’* [a medical advice app], *you know, and always make sure that we’re okay. Surveys were done, check-ins were done.”* (P5)

Some organizations recognized that not all employees were financially capable of providing their own tools of trade while working from home and therefore made provision for this:

*“They made sure I have a company cellphone as well as a laptop with enough data.”* (P6)

The organizational quality of empathy also went a long way for employees. Working from home brought about a new set of challenges. One participant pointed out that his organization was *“empathetic to family constraints resulting from the pandemic”* (P19).

During the lockdown, there were concerns around public transportation and whether passengers could ride safely. One participant detailed how their organization provided Uber rides*:*

*“So for the ones that traveled by taxi* [minibus taxi, a South African mass public transportation system], *just to make sure that we minimized contact with other people and not infect other colleagues, they organized e-hailing services for us and we didn’t have to pay anything for that, so that was from the company as well.”* (P15)

Leadership has typically been an important part of driving positive behavior from employees. To the participants, little acts of support from their leaders was recognized and appreciated during this time, as demonstrated by these quotes:

*“And I’ve also got a very understanding boss, where we have very open lines of communication. There are days where I’ve been very hard on myself and felt like I’m not delivering or I could do better, but she’s always very encouraging—when you do something she’ll give, like, the props, like, ‘Hey, well done, congratulations, keep up the good work,’ and it’s been quite the motivator.”* (P17)

*“I’m extremely grateful for it because I work in the city, my family, my children live in another province. I appreciated my line manager actually for providing me with that opportunity.”* (P20)

Similarly, colleagues played a pivotal role. One participant could not cope with the pressure of work and tending to their children during the lockdown and was grateful when colleagues who could assist did so:

*“I dropped the ball a few times during the lockdown, but some colleagues really stepped up for me.”* (P19)

Mothers felt especially hard hit by the lockdown given their new roles of employee, parent, teacher and coping was evidently difficult for them and thus needed all the emotional support they could get. Colleagues who are also moms understood this dynamic and were a source of this support for some which then brought them closer:

*“I feel like I have become closer to some colleagues who are also moms. Before the lockdown, we were colleagues, and now there are other colleagues whom I have upgraded to friend status. We talked about the difficulties of working from home during the lockdown. I am so grateful for them. They made me feel like I was not alone.”* (P17)

### Theme 3: Gratitude for a new way of working

The new way of working came as something of a relief for some. A few participants expressed relief that a hybrid work arrangement had been enforced in many organizations. They were grateful for the opportunity and ability to work from their own space, which allows for some flexibility. To them something that was long overdue for South Africa since it was already happening in some parts of the world. For many employees, after evaluating their finances against the new work arrangement, they believed they were saving time in traffic and money usually spent on fuel or public transport:

*“We were able to transition from what we knew from the normal nine-to-five basis in terms of going to work and being physically present to doing all that work in the comfort of your own space. To me, that is what I am grateful for—and being in the comfort of my own space.”* (P10)

*“I am grateful for the fact that companies realized that life can happen anywhere you are; you do not have to be in the office to do the work.”* (P12)

*“It could be early in the morning. It could be late at night having my laptop at home having access to Internet out always be attending to work related stuff. I work in a space where.”* (P20)

*“This pandemic has showed me I’m more efficient at home than I am in the office,”* (P21)

*“Spending money for transport I saved lots of money because I was working from home.”* (P7)

*“The first thing that stands out for me is not having to get up in the morning and sit in traffic to get to work.”* (P20)

It was hoped by them that new policies would be drafted by the organization which would allow for this to be a norm going forward. As the participants put it:

“*With this change also comes updated policies which ensure that this new configuration of work is here to stay for the foreseeable future. So our flexibility*… *[is] guaranteed for much longer.”* (P19)

The ability to work from home also afforded employees the opportunity to be more focused in their work, whereas the workplace has too many distractions.

*“I always say, you cannot work at work. There is far too much catching up and gossiping to be done with colleagues. I can go to work and come back home having done not a single ounce of work, whereas if I had just stayed at home, I would have ticked so many items off my to-do list. That balance is important, and I am happy it’s something we have that can enhance our productivity.”* (P19)

### Theme 4: Gratitude for the ability to put oneself first

A marker of the pandemic was how employees were stretched as they took on multiple roles. While under lockdown, many people had to be employees, parents, housekeepers, and teachers simultaneously. This put them under severe mental strain, as the boundaries between these roles had been completely broken down due to working and schooling from home. One participant recalled this challenge:

*“I found it very difficult to cope during this time. I am one of those people that is always ‘on’ regardless of the time of day. My work calls, I answer.”* (P19)

However, burnout made her rethink how she operates and forced her to do things differently.

*“I wish I had started much earlier in setting boundaries for myself. Just the power of ‘no’ has been liberating for me. When time for work ends, that’s it. That’s my time. That is my children’s time, and I do not feel guilty for not opening my mails. Even if I worked from home, there is a time to knock off and shut the laptop down. We will die working our fingers to the bone for these organizations, and my mental health is just too much of a sacrifice.”* (P12)

Working from home has afforded individuals to be able to take meaningful work breaks. Whilst at work, naptimes are forbidden, at home, a nap is both possible and easy as demonstrated by Participant 19 who states:

*“You can easily still get some rest because you have been in your own comfortable space.”* (P20).

This allows them to be more rested and more effective at work:

*“I am more rested that I didn’t have to get up so early in the morning and actually sit for hours just trying to get to work. So I yeah, I feel I was really”*. (P20).

### Theme 5: Gratitude for having resilience, optimism and spirituality as psychological buffers

As Participant 21 put it, she was *“grateful to be able to remain sane to an extent. But I think most of us lost it a bit.”* Coping with the effects of the pandemic would not have been possible had the participants not been able to lean on their positive outlook of the future. The participants were grateful for internal psychological buffers such as resilience which allowed them to bounce back from the difficult times. They also seemed optimistic and believing that they will overcome the obstacle that is the pandemic and as such, were grateful to not have been heavily burdened mentally by the pandemic. Believing in a higher power also helped them frame what was happening as something that God had control over, thus taking away the need to worry.

*“It’s to respect life and my colleagues that we are not here on our own consent; God brought us here”* (P11)

*“Yeah, my spirituality is what grounded me throughout this time. It was easy to look at the statistics”* (P8)

*“I’m pretty optimistic. I didn’t take much strain at work”* (P5)

*“It’s always made me realize to always look at the positive side of things and appreciate my work and the people I work with, instead of focusing more on the negative.* … *Looking into the positive just helps you come up with better solutions to resolve a problem.”* (P15)

Similarly, a shift in attitude provided some participants with the ability to see the pandemic differently and as such, they were grateful that they could therefore deal with the effects of the pandemic better and think about how to act differently in future to attain success in the new world:

*“We cannot change what we are going through, but we change our attitude toward it. I can cry all the time about how much this has impacted my ability to perform my job, or I can do the best I can with the cards I have been dealt. Optimism is a great buffer. It makes you feel like you can do all things. And I did.”* (P19)

*“I am grateful for the chance to rebuild. And also reevaluate. I think the underlining is I’m grateful for life.”* (P21)

## Discussion

The aim of the current study was to understand how employees experienced gratitude in the workplace during the pandemic.

Some participants in the present study experienced few or no negative life changes which meant that they kept their jobs, their homes, and did not contract COVID-19. These findings contradict those from a vast number of studies in South Africa and abroad that showed that when COVID-19 and its associated lockdowns hit, many were adversely impacted through a decline in wellbeing and job loss (Posel et al., 2021). Only a small minority felt no impact at all psychologically (cf. Kim et al., 2020); Ranchhod and Daniels (2020) suggest that individuals from lower socio-economic circumstances were disproportionally impacted economically. Given that most of our sample was employed in office jobs, they may have been better shielded from income loss which enabled them to maintain their pre-COVID-19 lifestyles and thus be grateful for their fortunes amidst the adversity faced by others around them.

The considerate nature of some of our participants’ workplaces was not lost on them. Human resource initiatives are often implemented as a strategic attempt to bring about positive organizational outcomes as was done by the employers of participants. Mariappanadar (2020) maintains that human resources can enhance employees’ lives, and perceived organizational support may decrease the health harm of work to employees—employees are of no productive help to their employers if they are in ill health. It is therefore a prerequisite for human resource management to play a role in minimizing the impact of crises and uncertainties on an organization’s talent, because this is seen in conjunction with organizational strategic decisions (Zagelmeyer and Gollan, 2012). An organization’s concern and care for its people is therefore as crucial a practice as any other strategic intent in the broader organizational strategy. With concern and care for its people, the organization stands to manage its effectiveness during crises. This is even more important during remote working and digitalization, as it offers the opportunity for human resource management to enhance and keep up productivity against these circumstances of change (Giguari, 2020). Giguari (2020) study showed that treating employees with compassion during the COVID-19 pandemic helped with the effective adaptation to new working practices (flexible working hours and remote work). Additionally, demonstrating organizational support can improve the wellbeing of employees—through emphasizing the value of individual strengths of employees, organizations can mitigate employee burnout and reduce the prevalence of mental illness for employees (Meyers et al., 2019). In this study, supervisor support seemed to be important and therefore recognized by the recipients. This is in keeping with Mihalache and Mihalache (2022) who found that one of the ways that employers can support their employees during environmental disruptions it to be accessible and communicate which in turn help employees cope in the same way our sample coped during the pandemic.

Our participants had the privilege of being able to switch from going physically into work to working remotely. In doing so, they were able to retain or even improve their productivity. This was in keeping with research by Wessels et al. (2019) who confirmed that flexibility in terms of remote working enhances productivity and performance. This means that working from home may have increased employees’ ability to meet their targeted performance metrics and possibly enhanced organizational productivity. Additionally, flexible work design has multiple benefits, such as enhancing the work–life balance, autonomy, and engagement of employees (Coenen and Kok, 2014).

Women have indicated a positive appreciation of the flexible work schedule for quite some time (Subramaniam et al., 2013). It is therefore noteworthy that flexibly designed workplaces were a positive experience during the COVID-19 pandemic for this group especially (Giguari, 2020). Past researched has demonstrated that flexible work schedules allow working mothers to enjoy the ability to continue working after having children, even in human-capital-intensive work (Chung and Van der Horst, 2018; Fuller and Hirsh, 2018). This demonstrates that work–life balance has been of utmost importance to women for a long time; the impact of the pandemic in terms of working from home has therefore only strengthened this.

Although working from home may have resulted in better work–life balance for some employees, it is noteworthy to indicate that it may have caused others to be engaged in work over and above normal working hours and possibly struggle to enjoy free time and social activities (Peters and Blomme, 2019). This may therefore necessitate conversations around employee wellbeing and wellness for the participants.

According to our findings, working from home also led to a significant reduction in household consumption, for instance, spending on transportation, electronics, clothing, and services. This finding is supported by Purwanto et al. (2020) who argued that working from home supports employees through flexible work schedules to complete work and save money usually spent on the work commute. This may have led to individuals’ experiencing improved financial health to some degree, a good motivation to be grateful.

The pandemic provided the opportunity for employees to focus on themselves. According to Clay (2020), being able to care for oneself during this time will allow individuals to enjoy a sense of wellbeing. For our participants, one of the ways this was achieved was through setting boundaries for themselves. This is supported by Hinton et al. (2020) who stated that saying “no” is an essential part of self-management. Further ways to self-manage can include behaviors such as identifying work and life priorities and then deciding what one should do now, what one can save for later and what can be delegated to competent others (Sekaja, 2021).

The study suggests that resilience, optimism and spirituality were key factors in overcoming the negative emotions associated with the burdens of the pandemic. Practicing virtuousness in the workplace has implications for desired actions and behavior. Chhajer et al. (2018) maintained that when employees exhibit increased levels of optimism, they tend to expect good things to occur in the future during a change. This optimism can also lead to positive performance outcomes (Cameron et al., 2011). A similar link to performance can be found with other virtuous acts such as support for others, forgiving mistakes, fostering meaningfulness, and showing gratitude. Since optimists are wired to expect good things to happen to them, they tend to exhibit more positive emotions and keep trying in the face of challenging circumstances (Seligman and Csikszentmihalyi, 2000); Riskind et al. (1996) maintain that optimism training interventions should not only act to diminish negative thoughts but also actually increase positive thinking for greater impact.

Resilience is conceptualized as the process of adapting positively to stress, threats, tragedy, or trauma (American Psychological Association, 2002). The sample in the study, having gone through COVID-19 and its effects, can therefore be said to be resilient. When individuals are resilient, they tend to experience less anxiety, negative emotion, and depression, and rather show greater life satisfaction, subjective wellbeing, and positive emotions (Wu et al., 2020), which may explain how the participants in this study were able to identify points of gratitude. Lastly, according to Daniel (2015), integrating spirituality into the workplace can be linked to lower levels of stress. Therefore, those with a spiritual grounding in our sample were able to buffer the effects of COVID-19 by drawing on their spirituality.

### Managerial implications

Gratitude is an internal feeling that can impact how employees view and interact with the world around them. Managers can try to cultivate this feeling by investigating the behaviors that elicit gratitude from their employees. These behaviors should be aligned to the employees’ values so that they care about what is being done for them. Understanding the behaviors that bring about gratitude may allow the employee to feel closer to their colleagues and more committed to the organization. After all, as Lee et al. (2019) argued, being a recipient of gratitude is indicative of a successful social interaction.

Expressing gratitude can be achieved in many ways. For example, Locklear et al. (2021) posited that the expression of gratitude can be achieved by reflecting and listing things for which one feels grateful. This intervention has been highly effective at improving wellbeing (Emmons and McCullough, 2003).

Managers should regularly and intentionally show gratitude to their employees for both big and small actions. This acts as a form of encouragement and motivation for the employees. A tangible way to demonstrate gratitude in the workplace is for managers to institutionalize the use of thank-you notes (Emmons and McCullough, 2003), even delivered by the manager to the employee’s workspace (Seligman et al., 2005), as a way of expressing gratitude to others. Research suggests, however, that gratitude letters have a shorter lasting positive effect than gratitude lists (see Wood et al., 2010).

In line with these ideas, this study suggests that managers can make use of these strategies to either elicit gratitude from or demonstrate gratitude to their co-workers and subordinates. This study also suggests that expressing gratitude in this way can help employees comprehend the positive impact they may have on their company of employment. Furthermore, fostering a culture of gratitude among employees can prompt individuals to feel a sense of connectedness to others (Wood et al., 2008a,b) and can motivate individuals to develop a sense of cohesion with respective others (Algoe, 2012). It therefore seems reasonable for managers to pre-emptively introduce interventions aimed at enhancing gratitude within the workplace to reap the associated benefits.

### Limitations

The study consisted of participants from a wide range of occupations. When it comes to the workplace, it could be argued that specific employment levels or jobs appreciate different gestures of kindness from their organization. For example, a worker on the lower end of the pay grade may be motivated by different things than a senior manager. The study therefore could have homed in on one of these specific groups for even more meaningful data. The study sought to investigate *what* working people were grateful for; to add to the value of the study, the participants could have been asked *how* their experience of gratitude enhanced their lives.

### Future directions

People of the world are now coming out of lockdowns and resuming life as they once knew it, or as close to that as possible, as they forge ahead with a new normal. To address the shortcomings found in this study, more research should be conducted using alternative approaches and designs. Future studies should employ a variety of sampling approaches with a larger sample of people from various occupations and demographic categories (e.g., race, gender, age, educational level, and seniority). This would help researchers understand how different employment categories experience gratitude by exploring the determinants and consequences thereof. It is an opportune time for researchers to understand how, after the height of the pandemic, employees experience gratitude and compare that to life during the pandemic. This is because there is a difference between experiencing life’s minor inconveniences (arguably the case before COVID-19) compared to potentially huge life changes (as a result of the pandemic), which would test any individual’s outlook on life. Future studies could look into whether workplace gratitude predicts wellbeing (or the reverse), using longitudinal and experimental approaches. Research yet to come could also look into whether being grateful at work has any drawbacks. For example, are workers who exhibit gratitude less inclined to address inequity or other injustices at their organization with its management? Furthermore, a study should be conducted on the impact of gratitude exercises among team members at work in order to understand which co-workers attributes are appreciated, how these can be harnessed and how these may impact professional relationships between team members. Lastly, there is no empirical evidence for the impact that a lack of gratitude has on the employee and the organization and this needs attention in order for scientists and organizations to understand the true value of gratitude, that is, not only what happens when employees are grateful, but also what may happen when they are not.

## Data availability statement

The original contributions presented in this study are included in the article/supplementary material, further inquiries can be directed to the corresponding author.

## Ethics statement

The studies involving human participants were reviewed and approved by Department of Industrial Psychology and People Management Research Ethics Committee. The patients/participants provided their written informed consent to participate in this study.

## Author contributions

LS conceptualized and supervised the study, analyzed the data, and prepared the manuscript for publication. KF, SM, CT, and LT carried out and completed the write up of the study that this manuscript in loosely based on. BM analyzed the data and helped prepare the manuscript for publication. MM and TM prepared the manuscript for publication. All authors contributed to the article and approved the submitted version.

## Conflict of interest

The authors declare that the research was conducted in the absence of any commercial or financial relationships that could be construed as a potential conflict of interest.

## Publisher’s note

All claims expressed in this article are solely those of the authors and do not necessarily represent those of their affiliated organizations, or those of the publisher, the editors and the reviewers. Any product that may be evaluated in this article, or claim that may be made by its manufacturer, is not guaranteed or endorsed by the publisher.
